# Clinical and neurophysiological characteristics of heterozygous *NPC1* carriers

**DOI:** 10.1002/jmd2.12059

**Published:** 2019-06-28

**Authors:** Alberto Benussi, Maria S. Cotelli, Valentina Cantoni, Valeria Bertasi, Marinella Turla, Andrea Dardis, Jessica Biasizzo, Rosa Manenti, Maria Cotelli, Alessandro Padovani, Barbara Borroni

**Affiliations:** ^1^ Neurology Unit, Department of Clinical and Experimental Sciences University of Brescia Brescia Italy; ^2^ Neurology Unit Valle Camonica Hospital Brescia Italy; ^3^ Department of Neuroscience, Psychology, Drug Research and Child Health University of Florence Florence Italy; ^4^ University Hospital “Santa Maria della Misericordia” Udine Italy; ^5^ IRCCS Istituto Centro San Giovanni di Dio Brescia Italy

**Keywords:** acetylcholine, cognition, executive functions, heterozygous, Niemann‐Pick disease type C, short latency afferent inhibition, transcranial magnetic stimulation

## Abstract

Niemann‐Pick disease type C (NPC) is an uncommon lysosomal storage disorder, which is characterized neuropathologically by cholinergic dysfunction and presents clinically with a broad series of neurological signs and symptoms. NPC is inherited as an autosomal recessive trait, caused by mutations in the *NPC1* or *NPC2* genes. However, recent reports have raised concerns on heterozygous *NPC1* gene mutation carriers, which historically have been considered as clinically unaffected, occasionally presenting with clinical parkinsonian syndromes or dementia. In the present study, we aimed at comprehensively assessing clinical, biochemical, and neurophysiological features in heterozygous *NPC1* gene mutation carriers. We assessed cholinergic intracortical circuits with transcranial magnetic stimulation, executive functions and plasma oxysterol levels in two families comprising two monozygotic twins with a homozygous *NPC1 p.P888S* mutation, four patients with a compound heterozygous *p.E451K* and *p.G992W* mutation, 10 heterozygous *NPC1 p.P888S* carriers, 1 heterozygous *NPC1 p.E451K* carrier, and 11 noncarrier family members. We observed a significant impairment in cholinergic circuits, evaluated with short‐latency afferent inhibition (SAI), and executive abilities in homozygous/compound heterozygous patients and heterozygous asymptomatic *NPC1* carriers, compared to noncarriers. Moreover, we reported a significant correlation between executive functions performances and both plasma oxysterol levels and neurophysiological parameters. These data suggest that heterozygous *NPC1* carriers show subclinical deficits in cognition, possibly mediated by an impairment of cholinergic circuits, which in turn may mediate the onset of neurological disorders in a subset of patients.

## 1 INTRODUCTION

Niemann‐Pick disease type C (NPC) is an uncommon autosomal recessive lysosomal storage disorder with accumulation of cholesterol and other lipid species caused by mutations of either the *NPC1* (95% cases)^1^ or *NPC2* genes,^2^ with considerable heterogeneity regarding biochemical, molecular and clinical features. Impaired cognitive functions have been reported in NPC patients, predominantly in executive functions, memory abilities, and visuo‐constructional skills.^3^, ^4^

Even though the precise functions of the proteins encoded by the *NPC1* and *NPC2* are yet to be entirely clarified, the disorder is denoted by the sequestration of unesterified cholesterol in lysosomes and late endosomes, with widespread effects on cholesterol cellular homeostasis.^5^, ^6^ Neuropathologically, an interesting parallelism has been observed between NPC and Alzheimer's disease, with both disorders sharing pathological features as the aggregation of amyloid‐β^7^ and tau tangles.^8^ This resemblance transcends the pathological aspects of the disease to neurophysiological measures, with the impairment of long‐term dependent (LTP)‐like synaptic plasticity and cholinergic dysfunction observed in both disorders.^9^, ^10^, ^11^, ^12^, ^13^

Historically, NPC has been considered as an autosomal recessive disease; nevertheless, several reports have now shown that heterozygous *NPC* carriers harbor subclinical abnormalities in cholesterol metabolism^14^, ^15^, ^16^ and in neuronal functions resulting in neurodegeneration.^17^, ^18^ Moreover, various heterozygous *NPC* mutations have been observed in patients with Parkinson's disease,^19^, ^20^ corticobasal syndrome and progressive supranuclear palsy.^21^ Just recently, an impairment of cholinergic circuits, evaluated with transcranial magnetic stimulation (TMS), has been observed in two heterozygous *NPC* carriers in a previously described family.^9^, ^22^ The generally accepted notion of NPC as a recessive disorder could therefore gain a different perspective.

To our knowledge, no evidence of subclinical deficits in cognitive domains, as executive functions, which are the initial and most relevant cognitive deficits in adult patients with NPC and extensively rely on cholinergic circuits,^23^, ^24^, ^25^ is currently available for heterozygous *NPC* carriers.

The objective of this study was (a) to evaluate the degree of cholinergic impairment using TMS and (b) evaluate subclinical cognitive dysfunctions in the executive domain in an extensive family of heterozygous *NPC* carriers compared to noncarriers and homozygous carriers.

## 2 METHODS

### 2.1 Standard protocol approvals, registrations, and patient consents

All procedures followed were in accordance with the ethical standards of the responsible committee on human experimentation (institutional and national) and with the Helsinki Declaration of 1975, as revised in 2000. Informed consent was obtained from all patients for being included in the study. The study protocol was approved by the local ethics committee (Brescia Hospital), #NP2745 approved 20.09.17.

### 2.2 Participants

**Figure 1:**
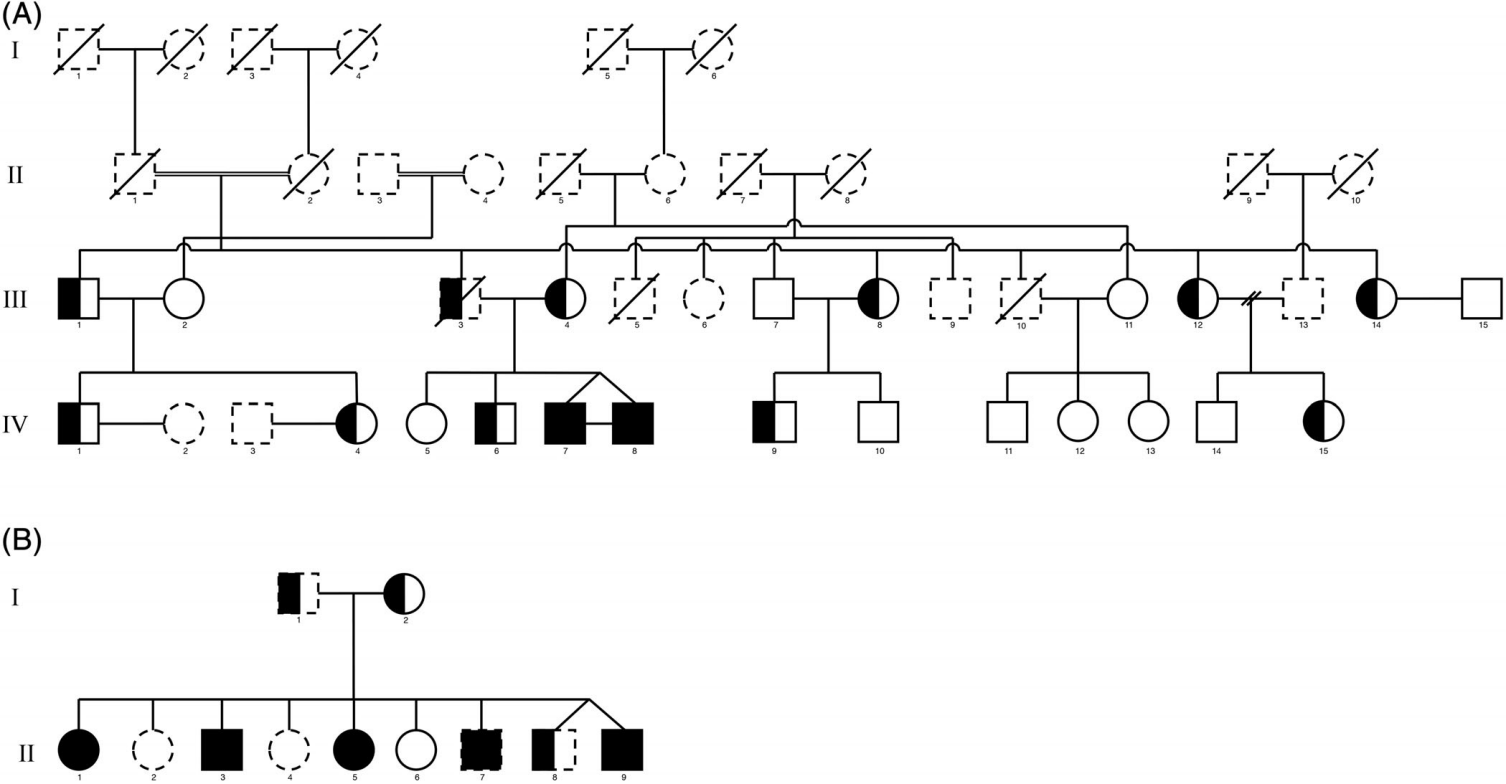
Pedigree of the NPC family considered in the present study. Participants with dashed lines were not evaluated in the present study

Twenty‐eight participants belonging to a previously described family^9^, ^22^ (Figure 1A) and to a novel family (Figure 1B), were included in the present study.

The following exclusion criteria were applied: (a) severe head trauma in the past, (b) history of seizures, (c) history of ischemic stroke or hemorrhage, (d) pacemaker, (e) metal implants in the head/neck region, (f) severe comorbidity, or (g) pregnancy.

### 2.3 Biochemical and molecular studies

Patients underwent molecular analysis of the *NPC1* gene, as previously described,^22^ and assessment of plasma oxysterols (cholestane‐3β,5α,6β‐triol) and 7‐ketocholesterol.^26^

### 2.4 Executive functions assessment

For the purpose of the present study, we aimed at assessing executive control abilities using a Flanker task^27^ and cognitive flexibility, using Stroop color‐word test.^28^

A revised version of the Flanker task was implemented in all patients.^27^ Stimuli were displayed sequentially on the center of the computer display. Every assessment began with the appearance of a fixation cross for 400 milliseconds (ms). Then, the stimulus was presented for 1700 ms followed by a variable interstimulus interval (1500‐2000 ms). Participants were taught to push the right or the left switch of a button box as rapidly as possible if the pointer displayed in the middle of the computer screen pointed to right of left, respectively. The central targets could be displayed along with four additional pointers flanked in the same orientation as the central one (← ← ← ← ←) (ie, congruent state) or flanked in the opposite orientation as the central target (→ → ← → →) (ie, incongruent state). Moreover, further neutral dashes (‐‐ → ‐‐) (ie, null state) could be displayed with the target. Correct responses and better performance (ie, higher precision and reduced reaction times) are observed with congruent flankers while conflicting information (ie, incongruent flankers) generally induce performance decline (ie, lower precision and greater reaction times). All trials were presented in a pseudo‐randomized order (32 for each condition, 96 total). Presentation software (www.neurobs.com) was implemented for stimulus presentation and data collection. The task lasted 6 minutes approximately without breaks.

Furthermore, we applied a short form of Stroop color‐word test.^29^ The participants were taught to read the color names (part A), name the ink colors of some circles (part B), and, eventually, identify the ink color of the printed color words (part C) as rapidly and as precisely as possible in all the three tasks. There was no time restriction to finish each task. An interference time measure was computed by deducting the average time required to finish the first two tasks from the time required to complete the third task and an interference errors measure was computed by deducting the mean number of errors recorded in the first two tasks from the errors shown in the third subtask. During the test, examiners did not point out errors made by the participants.

### 2.5 TMS assessment

To apply TMS, a figure‐of‐eight coil with a loop diameter of 70 mm, was connected to a Magstim BiStim^2^ system (Magstim Company, Oxford, UK), as previously reported.^30^ Surface Ag/AgCl electrodes positioned in a belly‐tendon montage on the right first dorsal interosseous muscle were used to record motor evoked potentials (MEPs), connected to a BIOPAC MP150 electromyograph (BIOPAC Systems Inc., Santa Barbara, California).

To investigate cholinergic circuits, we assessed short‐latency afferent inhibition (SAI) applying a conditioning‐test design, as previously reported.^31^, ^32^ The conditioning (ie, preceding) stimulus was characterized by 200 μs‐pulses of electrical stimulation applied to the median nerve at the wrist (right hand) through bipolar electrodes with the cathode targeted proximally, with an intensity directed at evoking a minimal visible twitch of the thenar muscles. The test (ie, following) stimulus was characterized by a TMS stimulus over the contralateral motor cortex, set at an intensity to evoke a MEP of roughly 1 mV in amplitude. Test stimuli were followed by conditioning stimuli at an inter stimulus interval (ISI) of −4, 0, +4, and +8 ms, timed to the latency of the N20 component of the somatosensory evoked potential, which was induced by stimulation of the median nerve at the wrist.

Ten stimuli were applied pseudo randomly for each ISI, while 14 for the test stimulus alone. The peak‐to‐peak amplitude of the MEPs preceded by a conditioning stimulus was reported as a percentage of the average unconditioned response. The time between different trials was set at 5 seconds (±10%).

Patients were instructed both by audio and visual feedback to maintain complete muscle relaxation throughout the experiment and, if the recorded data was deteriorated by the patients' movements, the whole protocol was restarted and the data discarded.

### 2.6 Statistical Analyses

Clinical, biological, and neuropsychological characteristics were compared with one‐way analysis of variance (ANOVA), two‐way mixed ANOVA, or the Fisher exact test, as appropriate. Reaction times and accuracy recorded in the Flanker task were analyzed using a two‐way mixed ANOVA with FLANKER CONDITION (congruent, incongruent, null) as within‐subject factor and GROUP (homozygous/compound heterozygous carriers, heterozygous carriers, noncarriers) as between‐subject factor, whereas Stroop task data were analyzed using a one‐way ANOVA with GROUP as between‐subject factor.

TMS measures were compared with a two‐way mixed ANOVA with ISI as within‐subject factor and group as between‐subject factor. If a significant main effect was obtained, group differences were examined with post hoc tests (Bonferroni correction for multiple comparisons). To check for sphericity violation, the Mauchly test was used.

Pearson's correlation was run to assess the relationship between neuropsychological and neurophysiological parameters.

For statistical analyses, SPSS version 21 (SPSS, Inc., Chicago, Illinois) was used.

## 3 RESULTS

### 3.1 Participants

Two monozygotic twins with a homozygous *c.2662 C>T(p.P888S)* mutation in the *NPC1* gene (18q11.2), 10 heterozygous *NPC1 p.P888S* carriers, four patients with a compound heterozygous *c.1351G > A (p.E451K)* and *c.2974G > T (p.G992 W)* mutation, one heterozygous *NPC1 p.E451K* carrier, and 11 noncarrier family members were included in the present study (see Figure 1). Demographic characteristics of included subjects are reported in Table 1.

**Table 1:** Demographic, clinical, and neurophysiological characteristics of included patients

Variable	Niemann‐Pick C patients	Heterozygous carriers	Noncarriers	P‐value
Number	6	11	11	‐
Age	26.3 ± 7.0	46.0 ± 12.3	39.7 ± 14.9	0.018^a^
Gender (Female) (%)	2 (33.3)	7 (63.6)	6 (54.5)	0.487^b^
Accuracy %				0.730^c^
Congruent	97.4 ± 4.2	98.6 ± 2.6	99.4 ± 1.3	‐
Incongruent	92.2 ± 5.8	94.0 ± 11.9	96.6 ± 5.0	‐
Null	98.4 ± 3.8	96.9 ± 5.0	98.8 ± 2.6	‐
Flanker test RTs				0.046^c^
Congruent	901.3 ± 50.5	728.5 ± 103.4	602.8 ± 73.2	‐
Incongruent	932.4 ± 60.3	856.4 ± 143.2	732.5 ± 124.6	‐
Null	861.4 ± 87.3	707.1 ± 128.6	558.8 ± 53.5	‐
Stroop test				
Time corrected	23.5 ± 7.7	20.3 ± 9.6	19.4 ± 6.6	0.618^a^
Errors corrected	0.0 ± 0.0	0.25 ± 0.5	0.27 ± 0.9	0.667^a^
TMS				
Mean SAI (0, +4)	0.92 ± 0.01	0.72 ± 0.07	0.46 ± 0.07	0.005^c^

*Note*: Results are reported as average ± SD unless otherwise specified.

Abbreviations: RTs, response times; SAI, short latency afferent inhibition; TMS, transcranial magnetic stimulation.

a One‐way ANOVA.

b Chi‐square test.

c Two‐way mixed ANOVA.

### 3.2 Biochemical analysis

Plasma biomarker levels were available for the *p.P888S* family (Figure 1A). For plasmatic oxysterols (cholestane‐3β,5α,6β‐triol), we observed only a nonsignificant difference in heterozygous carriers (29.56 ± 10.55 ng/mL) compared to noncarriers (29.05 ± 6.58 ng/mL) (reference values: 27.16 ± 5.48 ng/mL). For 7‐ketocholesterol we also observed a nonsignificant increase in heterozygous carriers (86.35 ± 45.12 ng/mL) compared to noncarriers (73.09 ± 20.29 ng/mL), with reference values of 67.47 ± 15.69 ng/mL.

### 3.3 Executive functions assessment

#### 3.3.1 Flanker task

Regarding accuracy data, at the two‐way mixed ANOVA we did not observe a significant FLANKER CONDITION (congruent, incongruent, null) **×** GROUP (homozygous/compound heterozygous carriers, heterozygous carriers, noncarriers) interaction (*F*(4,48) = 0.51, *P* = .730, partial *η* ^2^ = 0.04). We found a significant main effect for FLANKER TASK CONDITION (*F*(2,48) = 6.67, *P* = .003, partial *η* ^2^ = 0.22), but not for GROUP (*F*(2,24) = 0.61, *P* = .552, partial *η* ^2^ = 0.48). At post hoc tests, we observed a significant difference in Flanker test accuracy between congruent and incongruent conditions (*P* = .035), between incongruent and null conditions (*P* = .039), but not between congruent and null conditions (*P* = 1.000).

**Figure 2:**
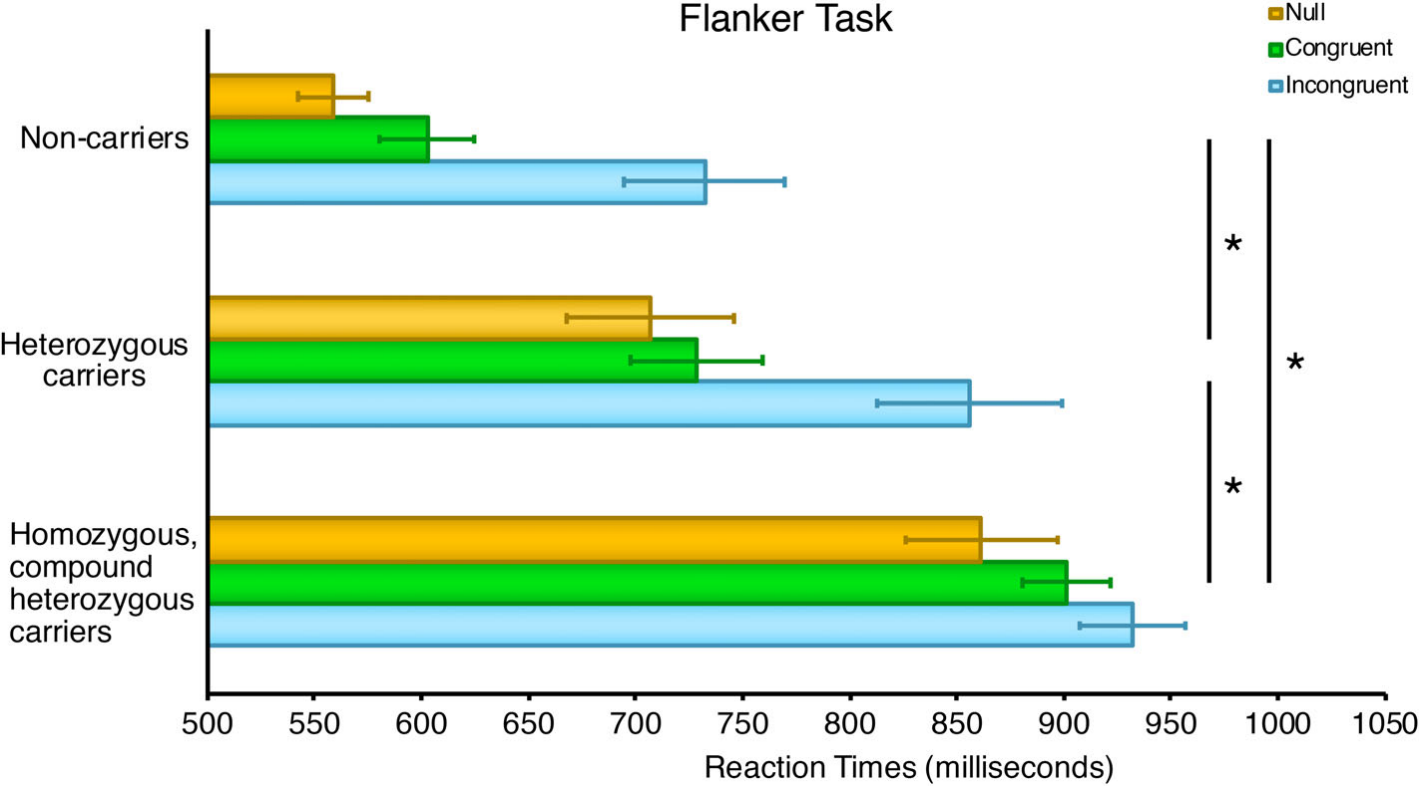
Average Flanker test response times (milliseconds) ± SE in homozygous/compound heterozygous and heterozygous *NPC1* mutation carriers compared to noncarriers. *Significant difference between groups (post hoc correction for multiple comparisons)

With regard to reaction times, at the two‐way mixed ANOVA we observed a significant FLANKER CONDITION **×** GROUP interaction (*F*(2,48)= 2.62, *P* = .046, partial *η* ^2^ = 0.18). We found a significant main effect for FLANKER TASK CONDITION (*F*(2,48)= 41.74, *P* < .001, partial *η* ^2^ = 0.63) and for GROUP (*F*(2,24) = 15.78, *P* < .001, partial *η* ^2^ = 0.42). At post hoc tests we observed longer reaction times in incongruent (*P* < .001) and in congruent conditions (*P* = .026) than in neutral conditions, and longer reaction times in incongruent than in congruent conditions (*P* < .001). Moreover, post hoc analyses recorded longer reaction times both in homozygous/compound heterozygous (*P* < .001) and heterozygous (*P* = .010) carriers as compared to noncarriers. Furthermore, homozygous/compound heterozygous carriers recorded longer reaction times than heterozygous carriers (*P* = .027). See Figure 2 for details. Flanker task reaction times and accuracy are reported in Table 1.

#### 3.3.2 Stroop task

No significant differences in the Stroop test were found. Stroop task scores are reported in Table 1.

### 3.4 Neurophysiological assessment

**Figure 3:**
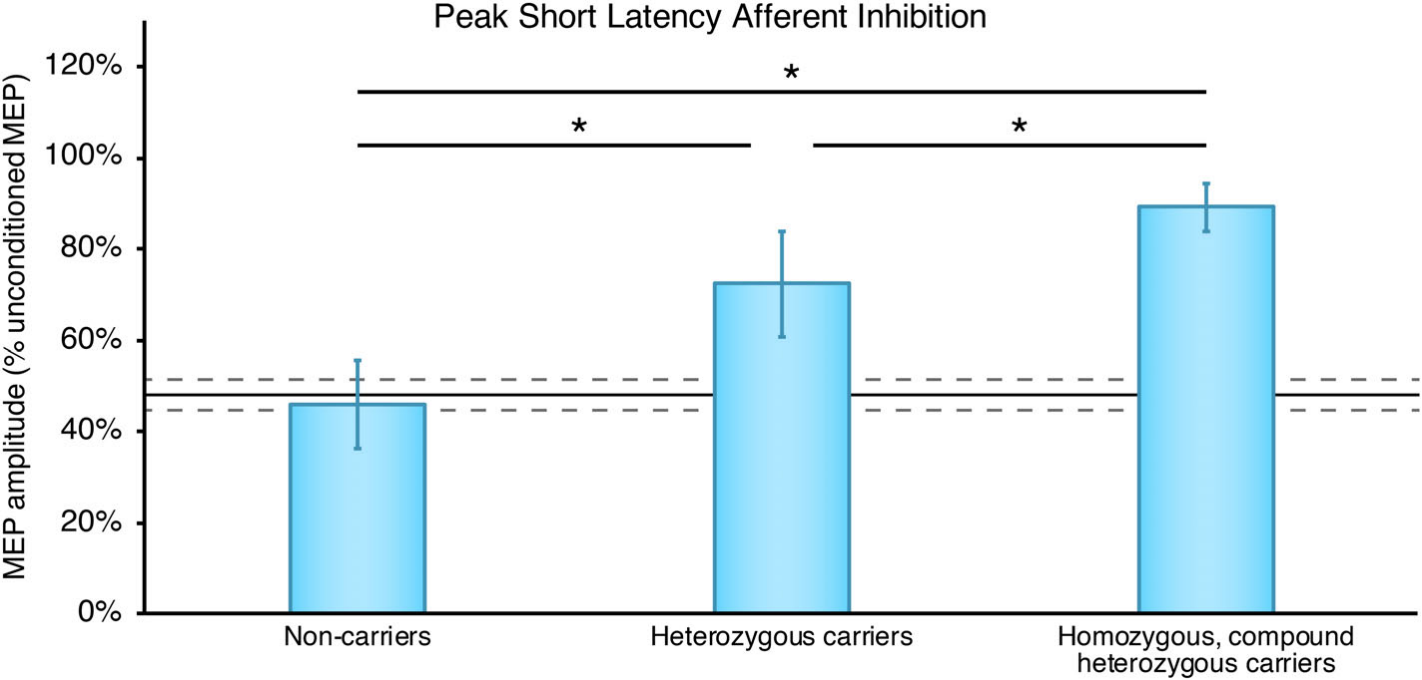
Average peak short latency afferent inhibition, marker of cholinergic neurotransmission, ±SD at ISI +4 ms, in homozygous/compound heterozygous *NPC1* carriers, heterozygous carriers and noncarriers. MEP: motor evoked potential. Black line: reference values for a group of healthy controls (n = 10) with SDs (gray dashed line). *Significant difference between groups (post hoc correction for multiple comparisons)

TMS was performed on 26 participants (two homozygous *P888S* and four compound heterozygous *p.E451K‐G992 W NPC1* carriers, 10 *P888S* and 1 *p.E451K* heterozygous carriers and nine noncarriers). Repeated measures ANOVA performed on SAI revealed a statistically significant ISI **×** GROUP interaction, *F*(6,69) = 3.47, *P* = .005, partial η^2^ = 0.23, with post hoc comparisons showing a difference between noncarriers and heterozygous carriers at ISI 0 and +4 (all *P* < .001), and between noncarriers and homozygous/compound heterozygous carriers at ISI +0 and +4 ms (all *P* < .001) and at ISI +8 ms (*P* = .027). Significant differences were also observed between heterozygous carriers and homozygous/compound heterozygous carriers at ISI +0 and +4 ms (all *P* < .005) (see Table 1 and Figure 3).

### 3.5 Correlations between biochemical, neuropsychological, and neurophysiological measures

We observed a significant positive strong correlation between 7‐ketocholesterol levels and congruent condition reaction times (*r* = .50, *P* = .034). For cholestane‐3β‐5α‐6β‐triol, we observed a significant positive strong correlation with congruent (*r* = .70, *P* = .001), incongruent (*r* = .59, *P* = .007), and null condition reaction times (*r* = .66, *P* = .001) and a negative strong correlation with accuracy in the congruent (*r* = −.55, *P* = .011), incongruent (*r* = −0.50, *P* = .024) and null conditions (*r* = −0.55, *P* = .011). We did not observe significant correlations between 7‐ketocholesterol or cholestane‐3β‐5α‐6β‐triol levels and Stroop task scores or SAI values.

For SAI, we observed a significant positive strong correlation for congruent (*r* = .75, *P* < .001), incongruent (*r* = .542, *P* = .004) and null condition reaction times (*r* = .70, *P* < .001), while we did not observe any correlation for accuracy and all conditions. We did not observe significant correlations between SAI and Stroop task scores.

## 4 DISCUSSION

The aim of the present work stemmed from the evidence that heterozygous carriers of *NPC1* gene mutations, which historically have been considered as clinically unaffected, occasionally develop neurological disorders such as extrapyramidal syndromes or dementia.^19^, ^20^, ^21^, ^33^ Thus, it might be postulated that either the neurological disorder and the heterozygous *NPC1* mutation occur independently, or that the heterozygous *NPC1* gene mutation might confer susceptibility to the development of a neurological disorders. If this latter were the case, the treatment approach in heterozygous *NPC1* mutation carriers cannot exclude a priori the current available treatments for NPC.

We comprehensively evaluated heterozygous *NPC1* gene mutation carriers belonging to two different families, and we compared biochemical parameters, executive abilities, and neurophysiological assessment with those of noncarrier family members.

We observed significant impairment of cholinergic circuits, along with selective executive function deficits in homozygous/compound heterozygous and heterozygous *NPC1* carriers compared to noncarriers.

From a biochemical viewpoint, heterozygous *NPC1* gene carriers have been shown to present with an intermediate phenotype regarding lipid metabolism and regulation, with cultured skin fibroblasts from *NPC1* heterozygotes showing an intermediate rate of production of cholesteryl esters, cholesterol esterification, and unesterified cholesterol storage levels compared to homozygous NPC and healthy controls.^16^ Moreover, the levels of 7‐ketocholesterol, plasma oxysterols and cholestane‐3β‐5α‐6β‐triol are significantly increased in human heterozygous *NPC1* carriers compared with those in healthy controls.^26^, ^34^, ^35^ However, in our study, we only observed a nonsignificant increase in plasma oxysterols, probably due to the small sample size, considering that we compared only 10 carriers and 10 noncarriers. Nevertheless, plasma oxysterols levels significantly correlated with deficits in executive functioning, further strengthening the link between plasma biomarkers and disease.

In heterozygous *NPC1*‐mutant mice, a significant loss of Purkinje cells and increase in brain cholesterol and hyperphosphorylated tau have been detected in the central nervous system.^17^ Parallelly, several case reports have described parkinsonism and Lewy body neuropathology in heterozygous *NPC1* carriers.^19^, ^20^, ^21^, ^33^

These findings were supported by the significant deficit in SAI circuits, here observed in patients with at least one *NPC1* mutation. SAI mainly reflects the integrity of cholinergic circuits, as it has been shown to be reduced after the administration of the acetylcholine antagonist scopolamine,^36^ to be increased after administration of acetylcholine‐esterase inhibitors that increase the availability of acetylcholine in the synaptic cleft^37^, ^38^ and to be impaired in patients with neurodegenerative disorders of the central cholinergic system, such as Alzheimer's disease and dementia with Lewy bodies.^39^, ^40^, ^41^, ^42^

In this view, the central nervous system regions that are regarded as most critical for attentional processing seem to be the frontal, prefrontal, parietal and somatosensory areas, where acetylcholine plays a crucial task in the top‐down control of attentional orientation and stimulus perception.^25^ This assumption is further supported by observations of NPC patients showing grey matter atrophy in cortical and subcortical structures that are involved in central cholinergic pathways.^43^, ^44^ These observations extend previous findings in heterozygous *NPC1* mutation carriers, in which deficits in neurophysiological parameters of cholinergic transmission and LTP‐like cortical plasticity have been reported.^9^, ^10^

Nevertheless, no subclinical dysfunctions in the executive skills have been so far systematically assessed in heterozygous *NPC1* carriers, while our executive function assessment provided support of the presence of subclinical difficulties in an executive control task in these individuals. We have already described deficits in the memory abilities, executive functions, and visuo‐constructional skills in the two monozygotic twins with homozygous *NPC1 p.P888S* mutation^9^, ^22^; herein, we further corroborated the previous reports by showing longer reaction times in the executive control task (ie, Flanker task) in these patients. The novel finding of the present study is that heterozygous *NPC1* mutation carriers displayed similar prolonged reaction times to an executive control task, thus suggesting the presence of subclinical difficulties in executive control abilities.

Heterozygous *NPC1* carriers have been usually regarded to be clinically unaffected, but it is still arguable whether *NPC1* haploinsufficiency can predispose *NPC1* carriers to intermediate and likely subclinical NPC expressions. We acknowledge that the present study presents some limitations: NPC is a rare disease, and our group of patients was relatively small, so clear‐cut associations need to be made with caution. Moreover, in the described family, consanguinity has been reported for some probands, implying possible co‐segregation of other disease traits other than *NPC* that may have an impact on the reported cognitive and neurophysiological measures. To overcome this limit, we included another family with a different mutation, obtaining comparable results. Future studies should also systematically assess other cognitive domains depending on cholinergic circuits, as working memory, episodic memory, and spatial memory function. Nevertheless, no significant alterations in these domains have been described in symptomatic NPC patients.^24^, ^25^

Taken together, our findings further corroborate previous evidence of subclinical deficits in executive control tasks and cholinergic dysfunction in heterozygous *NPC1* mutation carriers. Moreover, deficits in executive functioning tests significantly correlated with plasma oxysterol levels.

These results suggest that the assumption that heterozygous *NPC1* carriers are virtually unaffected may not always be correct, and consequently justifies further investigation, and clinical follow‐up is needed to further elucidate this issue. A subset of subjects with heterozygous *NPC1* gene mutation may be more susceptible to neurological disorders.

## CONFLICT OF INTEREST

Alberto Benussi, Maria Sofia Cotelli, Valentina Cantoni, Valeria Bertasi, Marinella Turla, Andrea Dardis, Jessica Biasizzo, Rosa Manenti, Maria Cotelli, Alessandro Padovani, and Barbara Borroni declare that they have no conflict of interest.

## AUTHOR CONTRIBUTIONS

A.B. and B.B. contributed to the conception and design of the study. A.B., M.S.C., V.C., V.B., M.T., A.D., R.M., M.C., A.P., and B.B. contributed to acquisition and analysis of data. A.B., A.D., and B.B. contributed to drafting the text and preparing the figures.
